# Engagement Opportunities in the NEXT STEPs Network

**DOI:** 10.1093/geroni/igaf122.453

**Published:** 2025-12-31

**Authors:** Cari Levy, Barbara Resnick, Joan Carpenter, Sarah Berry, Nicole Fowler

**Affiliations:** University of Colorado, Aurora, Colorado, United States; University of Maryland, Baltimore, Maryland, United States; University of Maryland School of Nursing, Baltimore, Maryland, United States; Hebrew SeniorLife & Harvard Medical School, Hebrew SeniorLife, Massachusetts, United States; Indiana University, Indianapolis, Indiana, United States

## Abstract

The Projects and Training Core strengthens research capacity by offering critical support to investigators through three initiatives: (1) Pilot Grants, which provide funding to both early-stage and experienced researchers to collect preliminary data essential for designing rigorous clinical trials; (2) An Experiential Learning Program, designed to introduce researchers new to nursing home research to the complexities of nursing home care. This program includes webinars, guided reflective discussions, and a hands-on, day-long visit to a nursing home to interact with staff and residents, fostering a deeper understanding of the environment; and (3) Research Studios, structured interactive sessions where researchers present their project ideas to an expert panel for constructive feedback and strategic guidance to refine study designs and methodologies. By providing these resources, training opportunities, and expert mentorship, the Projects and Training Core enhances the feasibility, quality, and impact of nursing home clinical trials. This initiative ensures that researchers are well-prepared to navigate the unique challenges of conducting research in nursing homes, ultimately advancing knowledge and improving care for residents.

